# The Effect of Emotion on Prosocial Tendency: The Moderating Effect of Epidemic Severity Under the Outbreak of COVID-19

**DOI:** 10.3389/fpsyg.2020.588701

**Published:** 2020-12-21

**Authors:** Yingying Ye, Tingting Long, Cuizhen Liu, Dan Xu

**Affiliations:** ^1^Department of Psychology, Zhejiang University of Technology, Hangzhou, China; ^2^School of Electronics Engineering and Computer Science, Peking University, Beijing, China; ^3^Department of Psychology, National University of Singapore, Singapore, Singapore

**Keywords:** COVID-19, positive emotion, negative emotion, prosocial tendency, big data

## Abstract

During the outbreak of COVID-19, information on the epidemic inundated people’s lives and led to negative emotions (e.g., tension, anxiety, and fear) in many people. This study aims to explore the effect of various emotions on prosocial tendencies during the COVID-19 outbreak and the moderating effect of the severity of the epidemic. We explore these effects by conducting a text analysis of the content of posts by 387,730 Weibo users. The results show that the severity of the epidemic promotes prosocial tendencies; anger motivates prosocial tendencies significantly; and the severity of the epidemic moderates the effects of three emotions—anger, sadness, and surprise—on prosocial tendencies. These findings provide a reference for exploring the positive significance of major disasters.

## Introduction

Since December 2019, the prevalence of the coronavirus disease 2019 (COVID-19) has grown exponentially. On January 20, 2020, the National Health Commission of the People’s Republic of China announced a comprehensive upgrade in the prevention and control of the epidemic. Since then, COVID-19 has attracted extensive attention in China with an enormous number of searches and discussions on social media platforms. During the COVID-19 outbreak, people’s lives have been inundated with epidemic-related information. Thus, it was inevitable that negative emotions such as tension, anxiety, and fear would arise in those affected by the epidemic (Bao et al., 2020). Simultaneously, news of more efficient measures to bring the epidemic under control coupled with touching stories of the medical staff reported in the media spread warmth and hope among the public. Under the influence of these emotions, various prosocial behaviors have been observed during the epidemic. For example, people worldwide have donated money or protective equipment to help prevent the spread of the virus. Thousands of medical staff have volunteered to travel to areas with severe outbreaks to assist with treatment. Prosocial behavior contributes to preventing the spread of an epidemic and improving people’s mental health (Yang et al., 2018). Furthermore, the public’s prosocial tendencies may reduce the social unrest caused by major disasters and help to maintain social stability.

From a macro perspective, we define prosocial tendencies as the degree of prosocial attitude or action in a given population that is then reflected on social network platforms. Although people who post about some prosocial acts or topics may be the sender or recipient of the prosocial act, we focused on words that reflect prosocial tendencies and extracted them from the post’s text. It is essential to explore the relationship between people’s multiple emotions and prosocial tendencies during this epidemic. The government and relevant agencies can then take this information and monitor the public’s emotions so as to regulate prosocial tendencies; they can do this by adjusting people’s emotions so as to promote groups’ prosocial tendencies and prevent the decrease of prosocial tendencies.

### Research on Disaster and Prosocial Behavior

Undoubtedly, the effects of COVID-19 amount to a human disaster. A large body of research suggests that trauma or disaster experience leads to a widespread increase in prosocial behaviors such as volunteering and/or donating money or other material goods and services (Frazier et al., 2013). For example, after the 9/11 terrorist attack, it was reported that 35–62% of undergraduates engaged in various volunteer behaviors such as donating blood, contributing money to help victims, and praying (Piferi et al., 2006). In a study on collective trauma, Hurricane Hugo victims reported more helping behavior than non-victims (Kaniasty and Norris, 1995). Additionally, Rao et al. (2011) found that the degree of prosocial behavior increased proportionately with increasing levels of residential devastation during the Wenchuan earthquake, the effect of which lasted for at least one year. When considering situational demands (Vollhardt, 2009), it appears that the more severe the disaster, the higher the number of opportunities and requirements to help others. Taking these findings together, Hypothesis 1 is that the severity of the epidemic increases prosocial tendencies.

### Research on Emotions and Prosocial Behavior

The relationship between emotions and prosocial behavior is complicated. Previous studies have primarily explored the relationship between positive/negative emotions and helping behaviors based on emotional valence (Forgas et al., 2008). Many studies have found that positive emotions promote prosocial behaviors. The meta-analysis results of Carlson et al. (1988) showed that a majority of positive emotion contributes to helping behaviors, while the impact of negative emotions on prosocial behavior remains controversial. For instance, anger motivates others’ prosocial behavior by making threats of malicious behavior (van Doorn et al., 2014). The influence of dispositional sadness and negative emotions such as anger on sympathy and prosocial behavior differs (Edwards et al., 2015). Lerner and Keltner (2000) proposed the Appraisal Tendency Framework (ATF) to explain the distinct effects of negative emotions: it posits that the influence of emotions on decision-making is reflected more in the types of emotions, rather than their valence. Through the appraisal tendency, inspired by its core evaluation subject, specific emotions affect individuals’ behavioral decisions (e.g., helping decisions). According to this framework, although both anger and sadness have a negative valence, appraisals of individual control of adverse events characterize anger and appraisals of situational control of negative events characterize sadness (Keltner et al., 1993; Lerner and Keltner, 2000). Considering the distinct roles of emotions in behavior, Hypothesis 2 is that emotions differently predict prosocial tendencies. Specifically, negative emotions such as sadness and anger have opposite influences on prosocial tendencies: sadness tends to lead to avoidance and negatively predicts prosocial tendencies, whereas anger is focused on external objects and tends to positively predict prosocial tendencies.

However, the Appraisal Tendency Framework does not clearly explain the mechanism of how emotions affect prosocial behavior; the Mood-Behavior Model (MBM) proposed by Gendolla (2000) further explains this process. The MBM posits that emotion mainly affects prosocial behavior by influencing behavioral preferences and interests based on a hedonic motive, the informational effects on behavior-related judgments and appraisals, and the interaction between the two. It is important to note that this theory is presented in the context of a non-threatening situation. Based on this, we will further explore the relationship between emotion and prosociality under the influence of COVID-19 and test the explanatory power of the Appraisal Tendency Framework and the Mood-Behavior Model under the condition of demand.

### Effect of Emotion on Prosocial Behavior Under the Influence of Disaster Severity

The phenomenon referred to as altruism born of suffering (ABS; Vollhardt, 2009) explains that encoding control moderates the relationship between negative emotions caused by suffering and prosocial behavior; this suggests a motivational modulation of prosocial behaviors (Vollhardt, 2009). The phenomenon referred to as required helpfulness arises in extreme situations of high stress and danger in which situational demands may trigger the motivation to help. Broadly, suffering implies situations in which people are required to help others (Southwick et al., 2005; Vollhardt, 2009). Based on the above theories, Hypothesis 3 is that the severity of the epidemic moderates the relationship between emotions and prosocial tendencies. The more severe the epidemic, the greater the relationship between emotions and prosocial tendencies.

### The Current Study

The current study aims to explore the effects of emotions and their interactions with the severity of the epidemic on prosocial tendencies during the COVID-19 outbreak. Six basic emotions (happiness, anger, sadness, fear, disgust, and surprise) proposed by Ekman (1992) and prosocial tendencies were assessed from the big textual data of Sina Weibo, the biggest and most popular public social media platform in China. Using text mining methods, researchers can explore the relationship between public emotions, prosocial tendencies, and the severity of the epidemic from a macro and comprehensive perspective. Text mining is a new research area in psychology that looks at the human mind and behaviors using web search data (Asur and Huberman, 2010; Wilson et al., 2012). At present, researchers have used the method to measure suicidal behavior, mental health, social prejudice, social inequality, and public responses to policies (Lai et al., 2017). When exploring psychological and behavioral characteristics in an epidemic situation, the strengths of big data mining are strong objectivity, high real-time, and ecological validity due to large sample size (Lai et al., 2017). In comparison, traditional questionnaire-based methods are time-consuming and small scale (Lai et al., 2017). Thus, big data methods are more suitable for this study.

## Materials and Methods

### Emotion and Prosocial Detection From Text

Detecting emotions from text is a task of computational linguistics. At present, academia has proposed a variety of technologies to accomplish this task. This study adopts a similar method to WordNet Affect presence discussed in Strapparava and Mihalcea (2008). This method judges the emotions communicated in a sentence based on whether it contains words in the emotion dictionary. Because this method is simple and effective, it is often used as a benchmark method to test the effectiveness of dictionaries (Staiano and Guerini, 2014) or compare newly proposed technologies (Rout et al., 2018). A similar method of measuring prosocial tendencies was used in this study (Frimer et al., 2014, 2015).

### Data Collection

In this study, the data were sourced from Sina Weibo. Specifically, COVID-19 related messages posted from January 20 to February 29, 2020 and containing the keyword “pneumonia” were obtained using web crawler technology. Initially, 745,153 Weibo posts by 411,235 users were gathered. After excluding the content from official verified accounts, 569,846 original messages posted by 387,730 users (37.4% male and 62.6% female) remained. Users’ identification markers were deleted and the posts were quantified through text analysis to carry out data de-identification. Since reposted Weibo content does not reflect a user’s own opinion, we retained only the comments attached to the repost. Also, we referred to the daily confirmed cases published by the National Health Commission of the People’s Republic of China as an index for the severity of the epidemic. All the posts and data used in the study were disclosed to the public (see Supplementary Materials). Ethical approval was obtained for this study.

### Experimental Materials

#### The Affective Lexicon Ontology

The Affective Lexicon Ontology, based on Ekman’s classification system of six basic emotions is commonly used in Chinese text emotion analysis (Ekman, 1992; Xu et al., 2008). The major dimensions of emotions—happiness, anger, sadness, fear, disgust, and surprise—were used in this study.

#### Prosocial Lexicon

Even though a useful dictionary containing prosocial words exists in the English language (Frimer et al., 2014, 2015), no such dictionary exists in Chinese at present. Therefore, we constructed a prosocial lexicon for this study. Prosocial behaviors cover a broad range of actions intended to benefit one or more people other than oneself, such as helping, comforting, sharing, and cooperating (Batson and Powell, 2003). Based on the definition of prosocial behavior, the first author collected words in the dictionary and literature related to prosocial behavior. Next, four undergraduate students majoring in psychology identified prosocial words from 2,441 messages on Weibo related to COVID-19 and then discussed to expand the previous word pool. For example, the word “lead” generally connotes negative influences and does not meet the prosocial definition exactly. Next, the four coders discussed the words in the word pool, and when up to 1/4 of the coders raise objections to a word, the word will be deleted. In total, 171 words remained in the pool at the end of the selection process. Subsequently, 10 senior undergraduate psychology students were invited to rate the extent to which these words exhibited prosocial tendencies on a 9-point Likert scale. The higher the score, the more prosocial the word. The inter-rater reliability for the rating was 0.78. After deleting words that were ranked low concerning prosocial associations (average score less than 6) and inconsistently among the 10 raters (standard deviation larger than 2), 155 words were retained to comprise the Prosocial Lexicon, including “dedication”, “volunteering”, “donation”, “help”, etc. Finally, each word’s mean score was mapped to a range of 1–9 using min-max normalization (see Supplementary Materials for details). We translated the prosocial lexicon into English and compared it with the Prosocial Word Dictionary in the Linguistic Inquiry and Word Count (LIWC) software tool. And we found that 73.55% of the words in our lexicon appeared in the LIWC Prosocial Word Dictionary (Frimer et al., 2014, 2015). On the whole, the Prosocial Lexicon constructed in this study was found to be valid.

### Data Processing and Statistical Analyses

The words in the lexicon were added to the custom dictionary in Jieba to improve the accuracy of segmentation, after which the Jieba package in Python (Sun, 2012) was used to segment the text to obtain words. Following this, the emotion and prosocial scores for each Weibo text were calculated. Specifically, the text was traversed and the frequency of each word in the lexicons was calculated. Next, the frequency of each word was weighed by its rating in the lexicons: for words in the prosocial lexicon, the ratings refer to the average ratings of prosociality of words obtained from raters; for words in the emotion lexicon, the rating is directly obtained from the emotion lexicon. The weighted frequency of each word was then accumulated to form the score of each dimension in prosocial tendencies and emotions. The weighted frequencies of words following negative words such as “rarely” and “not” were reversed before being accumulated. Finally, the daily average emotional scores and prosocial scores per Weibo were obtained. The daily average score is an indicator that reflects the prosocial tendencies and emotions per day. The higher the daily prosocial score, the stronger the prosocial tendencies. Similarly, the higher the emotion score of a particular dimension (e.g., happiness), the stronger the corresponding emotion. The number of days for the study was 41, and the number of Weibo posts participating in the calculation every day ranged from 847 to 18,364, with an average of 13,898.7. Next, these indicators were analyzed using SPSS ver. 26.0. The moderating model analyses were constructed using Hayes’s (2013) PROCESS macro (Model 1), with all variables standardized. The bootstrap method was used to test the significance of each effect and a robust standard deviation of parameter estimation was obtained (Hayes, 2013).

## Results

### Preliminary Analyses

The means, standard deviations (*SD*), and correlations for six basic emotions, prosocial tendency, and severity of the epidemic are reported in Table 1. The severity of the epidemic correlated positively and significantly with prosocial scores but not with any of the emotions. Among the basic emotions, only fear correlated negatively with prosocial scores.

**TABLE 1:** Descriptive statistics and correlations of variables (*N* = 41).

Variables	*M*	*SD*	1	2	3	4	5	6	7	8
(1) Epidemic severity	1929.68	2325.36	1							
(2) Prosocial scores	3.68	1.53	0.31*	1						
(3) Happiness	1.93	0.47	–0.06	–0.05	1					
(4) Anger	0.18	0.09	–0.17	0.26	0.40**	1				
(5) Sadness	0.99	0.28	0.24	0.11	0.07	–0.06	1			
(6) Fear	3.41	1.25	–0.25	−0.37*	0.09	0.31*	−0.40**	1		
(7) Disgust	2.92	0.49	–0.09	–0.10	0.48**	0.48**	0.18	0.23	1	
(8) Surprise	0.14	0.08	0.28	–0.06	–0.02	–0.03	0.20	–0.18	0.27	1

*p < 0.05. **p < 0.01. The data were analyzed using the daily average emotional scores, average prosocial scores, and newly confirmed cases. The total number of days is 41 (*N* = 41).

### Main Effects of Emotions and Epidemic Severity on Prosocial Tendencies

Multiple regressions were constructed to examine the influence of emotions and epidemic severity on prosocial tendencies. Each emotion was analyzed independently. Specifically, each specific emotion and epidemic severity was entered first as an independent predictor of the prosocial score in the regression. Next, a product term of the two predictors was entered. Both emotion and prosocial scores were standardized before forming the product term (Aiken and West, 1991). The results of each regression formulation are presented in Table 2.

**TABLE 2:** The results of the six moderate regressions (*N* = 41).

Model	Dependent variable	Independent variable	*R*^2^	*F*	*B*	*SE*	*t*	*p*	95%CI
Model 1	Prosocial scores	Intercept	0.12	1.66	–0.02	0.15	–0.11	0.914	[*−*0.33, 0.29]
		Happiness			–0.09	0.17	–0.56	0.579	[*−*0.44, 0.25]
		Epidemic severity			0.26	0.16	1.64	0.110	[*−*0.06, 0.59]
		Happiness × Epidemic severity			–0.27	0.28	–0.96	0.341	[*−*0.84, 0.30]
Model 2	Prosocial scores	Intercept	0.28	4.74**	0.13	0.15	0.85	0.399	[*−*0.18, 0.44]
		Anger			0.45	0.16	2.92	0.006	[0.14, 0.77]
		Epidemic severity			0.79	0.25	3.13	0.003	[0.28, 1.30]
		Anger × Epidemic severity			0.77	0.38	2.02	0.050	[0.00, 1.53]
Model 3	Prosocial scores	Intercept	0.25	4.21*	0.13	0.15	0.91	0.369	[*−*0.17, 0.44]
		Sadness			–0.12	0.16	–0.75	0.460	[*−*0.43, 0.20]
		Epidemic severity			0.70	0.20	3.42	0.002	[0.28, 1.11]
		Sadness × Epidemic severity			–0.57	0.21	–2.79	0.008	[*−*0.99, *−*0.16]
Model 4	Prosocial scores	Intercept	0.19	2.94*	–0.04	0.16	0.22	0.831	[*−*0.30, 0.37]
		Fear			–0.26	0.19	–1.37	0.180	[*−*0.64, 0.13]
		Epidemic severity			0.24	0.15	1.54	0.132	[*−*0.07, 0.54]
		Fear × Epidemic severity			0.14	0.30	0.48	0.634	[*−*0.46, 0.75]
Model 5	Prosocial scores	Intercept	0.13	1.85	0.03	0.15	0.20	0.845	[*−*0.28, 0.34]
		Disgust			–0.01	0.16	–0.07	0.942	[*−*0.34, 0.32]
		Epidemic severity			0.42	0.19	2.26	0.030	[0.04, 0.80]
		Disgust × Epidemic severity			0.36	0.32	1.12	0.269	[*−*0.29, 1.00]
Model 6	Prosocial scores	Intercept	0.33	6.21**	0.17	0.14	1.21	0.233	[*−*0.12, 0.46]
		Surprise			–0.11	0.14	–0.79	0.433	[*−*0.40, 0.17]
		Epidemic severity			0.91	0.21	4.28	< 0.001	[0.48, 1.33]
		Surprise × Epidemic severity			–0.62	0.18	–3.46	0.001	[*−*0.99, *−*0.26]

*p < 0.05. **p < 0.01. The data were analyzed using the daily average emotional scores, average prosocial scores, and newly confirmed cases. The total number of days is 41 (*N* = 41).

The effect of the severity of the epidemic was statistically significant in four out of six regression models, namely anger, sadness, disgust, and surprise. The severity of the epidemic positively correlated with prosocial tendencies in all six emotion regression models.

Concerning the effect of emotions on prosocial tendencies, only anger was significant among the six basic emotions. Anger positively predicted prosocial scores. Other negative emotions (e.g., sadness, fear, and disgust), positive emotions (happiness), and surprise had no effect on prosocial scores.

### The Moderating Role of Epidemic Severity on the Relationship Between Emotions and Prosocial Tendencies

**FIGURE 1:**
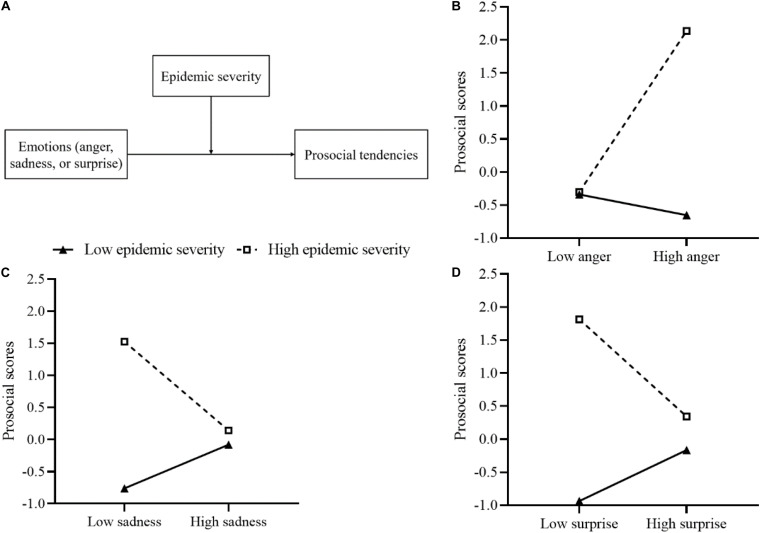
**(A)** The moderating model of the influence of emotions on prosocial tendencies. **(B)** The effects of epidemic severity on the relationship between anger and prosocial tendencies. **(C)** The effects of epidemic severity on the relationship between sadness and prosocial tendencies. **(D)** The effects of epidemic severity on the relationship between surprise and prosocial tendencies. **(B–D)** The severity of the epidemic was divided into high (M + 1SD) and low groups (M–1SD, or the minimum score).

Figure 1A presents the influence of emotions (anger, sadness, surprise) on prosocial tendencies, with the severity of the pandemic as a moderator. The interaction between emotions (anger, sadness, or surprise) and the severity of the epidemic on prosocial tendencies was significant, suggesting that the severity of the epidemic moderated the impact of these three emotions on prosocial tendencies. Simple slope analysis was used to analyze further the moderating mechanism of epidemic severity on these emotions. We divided the severity of the epidemic into high and low groups according to *M* ± *1SD* [high group = *M* + *1SD*, low group = *M−1SD* or the minimum score of daily newly confirmed cases (77)], to examine the specific effects of anger, sadness, and surprise on prosocial scores at different severity levels of the epidemic.

In the prediction model for anger, both the main effect of anger and the severity of the epidemic were significant. The interaction term of anger and epidemic severity positively predicted prosocial scores, indicating that the severity of the epidemic had a moderating effect on the impact of anger on prosocial tendencies. As depicted in Figure 1B, simple slope analyses showed that the relationship between anger and prosocial scores was significant at a high level of epidemic severity (*simple slope* = 1.22, *t* = 2.62, *p* = 0.013), and non-significant at a low level of epidemic severity (*simple slope* = *−*0.16, *t* = *−*0.57, *p* = 0.574).

In terms of sadness, the interaction term of sadness and epidemic severity significantly and negatively predict prosocial tendencies, which suggested that the severity of the epidemic played a moderating role in the effect of sadness on prosocial tendencies. As depicted in Figure 1C, sadness had a significant negative effect on prosocial scores when the severity of the epidemic was high (*simple slope* = *−*0.69, *t* = *−*2.31, *p* = 0.027), while no such effect was found when the epidemic severity was low (*simple slope* = 0.34, *t* = 1.87, *p* = 0.069). Interestingly, concerning the effects of two negative emotions, anger predicted prosocial tendencies in the opposite direction to sadness. The effect of anger on prosocial scores differed from that of sadness under diverse epidemic severity conditions.

Unexpectedly, the main effect of epidemic severity was significant, while the effect of surprise on prosocial scores was not. However, the interaction between surprise and epidemic severity negatively and significantly predicted prosocial tendencies, with epidemic severity having a moderating influence on the effect of surprise on prosocial tendencies. As depicted in Figure 1D, the relationship between surprise and prosocial scores was negative when the severity of the epidemic was high (*simple slope* = *−*0.74, *t* = *−*3.39, *p* = 0.002), but this relationship became non-significant when the severity of the epidemic was low (*simple slope* = 0.39, *t* = 1.83, *p* = 0.075).

## Discussion

In this study, we investigated the impact of emotions on prosocial tendencies and the moderating role of the severity of the epidemic. We did so by analyzing Weibo text data.

The correlation analysis demonstrates that the more fear people feel, the less prosocial they are. Excessive fear may weaken an individual’s empathy toward others and hinder the generation of prosocial behaviors (Eisenberg, 2002). Nevertheless, six emotions were not significantly correlated with the severity of the epidemic. The results show a significant main effect of epidemic severity on prosocial tendencies, suggesting that the increased severity of the epidemic motivates prosocial tendencies. As the epidemic becomes more severe, the prosocial tendencies of people with different emotions increase. Since the outbreak of the COVID-19 epidemic, many deeds of assistance have emerged. For example, people all over the country donated money and protective equipment to help fight the virus. Community volunteers spontaneously transported supplies and took care of the children of medical staff. Psychological counselors offered online psychological assistance to people who had experienced trauma or lost loved ones. As per the Altruism Born of Suffering (Vollhardt, 2009), people exhibit more prosocial behaviors in disaster situations. This is reflected in the case of the COVID-19 epidemic where prosocial tendencies increased significantly in response to the demand for materials such as protective equipment. This result supports Hypothesis 1—that the severity of the epidemic increases prosocial tendencies and is similar to the findings of Rao et al. (2011) concerning prosocial behavior in the aftermath of an earthquake.

Anger had a significant main effect on prosocial tendencies as it positively forecasted the public’s overall prosocial tendencies during the epidemic. The results indicate that the angrier people are, the higher their tendencies toward prosocial behavior. This finding is consistent with those of previous studies. For instance, the threat of malicious behavior following anger motivated prosocial behavior (e.g., cooperation) in others (van Doorn et al., 2014). According to the Mood-Behavior Model (Gendolla, 2000), angry individuals may blame the outbreak of the epidemic on others and the environment. These angry individuals become more involved in world events and pay more attention to the development of the group to which they belong, thus inspiring an increase in prosocial tendencies. Lv (2017) found that the greater the anger from inter-groups threat, the higher the inclination toward extreme pro-group behaviors. In other words, when the anger felt by an individual is caused by the threat of harm to the group to which they belong, it is more likely to inspire prosocial motives. Conversely, when a threat is directed at individuals rather than at groups, personal anger negatively predicts extreme pro-group behavior (Lv, 2017). In line with this claim, COVID-19 has placed society and even humankind in danger and therefore has evoked more prosocial tendencies that point to in-group members.

Moreover, the effect of anger on prosocial tendencies was moderated by the severity of the epidemic. Specifically, a positive association between anger and prosocial tendencies only exists when the epidemic severity level is high. When the epidemic worsened, many newly confirmed cases and deaths made individuals feel under significant threat. The combined effect of anger and threat resulted in individuals’ inclination to empathize, learning about the threat posed by the potentially dangerous environmental events, and engaging in prosocial behaviors (Silk and House, 2011). This finding further demonstrates that anger can be used to elicit prosocial behavior to counterbalance the disadvantageous position in which victims find themselves (Iyer et al., 2007; van Doorn et al., 2017).

The results suggest that sadness is a significant negative predictor of prosocial tendencies at a high level of epidemic severity. The experience of sadness included people’s appraisal of unpleasantness and barriers; appraisal included a feeling of loss of control that is central to sadness (Ellsworth and Smith, 1988). Conditions resulting from a high level of epidemic severity threatened people and the resulting increase in sadness was associated with lower levels of prosocial tendencies (Potts et al., 1989). Sadness reduced the performance of individuals’ attention tasks or narrowed their attention span, and made them more self-focused (Albert et al., 2010). When individuals are sad and too focused on their internal situation, prosocial behavior may be inhibited by over-arousal of emotions in the first place (Wakslak et al., 2007). This result is consistent with the attention focus pattern in which the pessimistic mood of people who are sunk in self-absorption is likely to reduce helping behavior (Rosenhan et al., 1981).

It is worth noting that the two negative emotions, anger and sadness, had opposite effects on prosocial tendencies. At a high level of epidemic severity, anger predicted prosocial tendencies positively and significantly while sadness predicted prosocial tendencies in a significantly negative direction. This result supports Hypothesis 2—that emotions differently predict prosocial tendencies. These findings demonstrate that people’s prosocial tendencies are determined by specific types of emotions, rather than a positive or negative emotional valence (Lerner and Keltner, 2000).

However, our findings that anger positively predicts prosocial tendencies and that sadness negatively predicts prosocial tendencies differ from the findings of previous research. For example, Kandrack and Lundberg (2014) found that sad individuals, compared to angry individuals, donated more money to individuals in a neutral social condition. In an emotional autobiographical memory task, Small and Lerner (2008) found that the group with sadness was more prosocial than the angry group: the sad group tended to support welfare policies with lower eligibility standards so that more people could receive government assistance. Keltner et al. (1993) purported that sad people were more inclined than angry people to attribute causality to situational factors. However, it must be noted that this study’s conditions differ from those of previous studies. The anger and sadness in previous studies were mostly induced in a laboratory setting that may lack ecological validity. Prosocial behavior measurements were also limited to a few categories (e.g., donations and welfare formulation; Small and Lerner, 2008; Kandrack and Lundberg, 2014). The context of our study was based on a real-life epidemic and the big data mining method was used to ensure ecological validity (Lai et al., 2017). Interestingly, although our study found that the relationships between sadness/anger and prosocial tendencies were not significant at a low severity level, the predicted directions were the same as in previous studies wherein sadness contributes to but anger hinders prosocial tendencies (Small and Lerner, 2008; Kandrack and Lundberg, 2014).

The basic emotion surprise may be positive or negative in different contexts (Alm et al., 2005). This study found that when the severity of the epidemic was high, surprise negatively predicted prosocial tendencies. As the epidemic became severe, the environment posed a significant threat to people. Thus, when people thought that their ability to control the situation was lower than expected, they were surprised and this over-arousal emotion may have inhibited helpfulness (Fabes et al., 1993). Moreover, the surprise emotion may have induced individuals to focus their attention on their own situation. As per the informational mood impact in Attention Focus Patterns (Rosenhan et al., 1981) and the Mood-Behavior Model (Gendolla, 2000), surprise is less likely to produce prosocial behavior when individuals pay more attention to themselves than to others. However, this finding contradicts Exley and Petrie’s (2018) finding that a request for a donation that was expected instead of surprising could decrease prosocial behavior. Notably, the element of surprise in Exley and Petrie’s study took place in a normal societal context where people could freely decide whether to help, whereas the element of surprise in our study occurred in the more dangerous context of the COVID-19 epidemic where the desperation of people made prosocial tendencies imperative. Thus our findings differ (Exley and Petrie, 2018).

In brief, this study found that the interaction between emotions (e.g., anger, sadness, and surprise) and the severity level of the epidemic on prosocial tendencies is significant, thus supporting Hypothesis 3—that the severity of the epidemic moderates the relationship between emotions and prosocial tendencies. Although anger and sadness are both negative emotions, they play reverse roles. Only when the severity level of the epidemic is high is the effect of the three emotions (anger, sadness, and surprise) on prosocial tendencies significant. In contrast, their impact is not significant at a low level of severity. According to Attention Focus Patterns and the Mood-Behavior Model, this may be because people feel more threatened in urgent and dangerous situations and this threat affects people’s attention (Bradley, 2009), which in turn affects the path of emotional influence on prosocial tendencies (Rosenhan et al., 1981; Gendolla, 2000).

In summary, our findings show that during the COVID-19 outbreak the effect of emotions (e.g., anger, sadness, and surprise) on prosocial tendencies differed from the findings in previous studies (Small and Lerner, 2008; Kandrack and Lundberg, 2014; Exley and Petrie, 2018). The results may be due to different research conditions. Most of the previous studies were conducted in everyday situations similar to a situation with a low level of epidemic severity and emotions (e.g., anger, sadness, and surprise) studied were found to influence prosocial behavior (Small and Lerner, 2008; Kandrack and Lundberg, 2014; Exley and Petrie, 2018). However, our study was conducted during a high level of epidemic severity. In this crisis and life-threatening situation, the effects of negative emotions (anger, sadness, fear, disgust) on prosocial tendencies are more complex and predict prosocial tendencies in different directions and to different degrees. Our study differs from the study of Forgas et al. (2008), which explains the relationship between emotion and behavior from the valence dimension. However, our results support Appraisal Tendency Framework: the effect of valence on behavior is uncertain and emotions of the same valence may have different effects on prosocial tendencies (Lerner and Keltner, 2000).

This study has some limitations that can be addressed in future studies. First, as with most text mining research, the current study used the “bag of words” method when processing text, which results in some loss of useful information (Qin et al., 2016). Second, as our prosocial lexicon was established in the context of the epidemic, the words it contains need to be specified in the future to make the lexicon more widely applicable. For example, it is useful to identify the prosocial tendencies of words as actions or intentions, as senders or recipients, and the agent as an individual or group. Third, the prosocial tendencies in this study are related more to in-group behaviors than out-group behaviors. It is a challenge of future research to distinguish prosocial tendencies between the in-group and the out-group individual. The basis for dividing groups may include the diagnosis status of COVID-19 and so on. Fourth, our prosocial tendency variable reflects prosocial behavior to a certain extent, but there is still difference between them. Besides, methods to measure prosocial tendencies or behaviors also differ and may not be directly comparable. Subsequent studies on prosocial behavior could be conducted using experimental methods to verify our results. For example, researchers could investigate the effect of emotions on prosocial behavior in both normal and threatening conditions.

This study elucidates the influence of emotion on prosocial tendencies during the severe phase of the COVID-19 epidemic. Based on our findings, we offer the following recommendations to people:

Concerning individuals, if and when severe major crises, like the COVID-19 epidemic, occur, people should pay closer attention to their anger, sadness, surprise, and other emotions because these emotions can help to perceive a situation sensitively and to absorb. Concerning anger, when people are angry during the outbreak of COVID-19 epidemic, they are advised to put more focus on the outside world, focus on the community’s requirements, and promote prosocial tendencies. Concerning the emotions of sadness and surprise, people should not pay too much attention to themselves and instead redirect these emotions toward others in a positive way. This will also decrease the negative impact of these emotions on themselves and others.

Concerning society at large, our findings provide insights into the public’s psychological and behavioral states during a major crisis and a scientific basis for public policy formulation.

## Data Availability Statement

The original contributions presented in the study are included in the article/Supplementary Material, further inquiries can be directed to the corresponding author/s.

## Author Contributions

YY contributed to conceptualization, methodology, formal analysis, investigation, visualization, project administration, writing-original draft, and writing-review and editing. TL contributed to data curation, investigation, software, writing-original draft, and writing-review and editing. CL contributed to conceptualization, methodology, supervision, and writing-review and editing. DX contributed to conceptualization, funding acquisition, resources, supervision, and writing-review and editing. All authors contributed to the article and approved the submitted version.

## Conflict of Interest

The authors declare that the research was conducted in the absence of any commercial or financial relationships that could be construed as a potential conflict of interest.
